# Predictors of prostate cancer screening among African American men treated at an Academic Medical Center in the Southern United States

**DOI:** 10.25082/CCR.2021.01.003

**Published:** 2021-04-02

**Authors:** Steven S. Coughlin, Deepak Nag Ayyala, John S. Luque, Justin Xavier Moore

**Affiliations:** 1 Department of Population Health Sciences, Medical College of Georgia, Augusta University, Augusta, GA, USA; 2 Institute of Public and Preventive Health, Augusta University, Augusta, GA, USA; 3 Institute of Public Health, Florida Agricultural and Mechanical University, Tallahassee, FL USA

**Keywords:** African Americans, men, prostate cancer, prostate specific antigen test, screening

## Abstract

**Background:**

The controversy surrounding prostate cancer screening, coupled with the high rates of incidence and mortality among African American men, increase the importance of African American men engaging in an informed decision-making process around prostate cancer screening.

**Purpose:**

To examine predictors of prostate cancer screening via the prostate-specific antigen (PSA) test. Secondary objectives were to examine whether African American men have been screened for prostate cancer; their confidence in making an informed choice about whether PSA testing is right for them; and whether they have talked with their provider about PSA testing and engaged in an informed decision-making process around prostate cancer screening.

**Methods:**

We conducted a study among a sample of African American men patients ages ≥ 40 years.

**Results:**

A total of 65 men completed the questionnaire (response rate = 6.5%). The mean age of the men was 64.4 years. Most of the participants (90.8%) reported a regular healthcare provider and that their provider had discussed the PSA test with them (81.3%). About 84.1% of the men ever had a PSA test, but only 38.0% had one in the past year. Most of the men reported that they make the final decision about whether to have a PSA test on their own (36.5%) or after seriously considering their doctor’s opinion (28.6%). About 31.8% of the men reported that they share responsibility about whether to have a PSA test with their doctor. About half of the participants (49.2%) reported that they have made a decision about whether to have a PSA test and they are not likely to change their mind. The majority of the men (75%) perceived their risk of prostate cancer to be about the same level of risk as other men who were their age. The men’s knowledge of prostate cancer was fair to good (mean prostate cancer knowledge scale = 10.37, SD 1.87). Knowledge of prostate cancer was positively associated with receipt of a PSA test (p < 0.0206).

**Discussion:**

The modest overall prostate cancer knowledge among these participants, including their risk for prostate cancer, indicates a need for prostate cancer educational interventions in this patient population.

## 1 Introduction

Prostate cancer is the most commonly diagnosed male cancer in the U.S. [1] African American men have the highest prostate cancer incidence rates in the world and the highest prostate cancer mortality rate of any racial/ethnic group in the U.S. [2] African American men are more likely to have locally advanced or metastatic prostate cancer at diagnosis, present at an earlier age, and have suboptimal outcomes to standard treatments [3]. African American men are 2.5 times more likely to die from prostate cancer than white men [1].

African American men are more likely to develop aggressive prostate cancer, yet less likely to be screened despite guidelines recommending shared decision-making about prostate cancer screening and PSA testing [1,3]. It is unclear whether screening through PSA testing reduces mortality [4]. However, no firm conclusions about the benefits-to harm ratio of PSA screening can be drawn in African American men due to their limited representation in two landmark clinical trials of the effectiveness of prostate cancer screening in reducing mortality from the disease [1]. A more recent study that used Surveillance, Epidemiology, and End Results (SEER) data to investigate survival disparities between African American and white men provided a compelling case for continued PSA testing for African American men [5]. The controversy surrounding prostate cancer screening, coupled with the high rates of incidence and mortality among African American men, make it that much more important for African American men to engage in an informed decision-making process around prostate cancer screening [6]. Previous studies have suggested that men who are more knowledgeable about prostate cancer are more likely to have been screened [7]. While informed decision-making is the current recommendation for prostate cancer screening, recent studies indicate that many African American men may not be making informed decisions about prostate cancer screening [1]. This is partly due to patients having limited knowledge of prostate cancer screening and African Americans being more likely to have inadequate health care.

We conducted a study of a sample of male, African American patients ages ≥ 40 years to examine predictors of prostate cancer screening. Secondary objectives were to examine whether African American men have been screened for prostate cancer; their confidence in making an informed choice about whether PSA testing is right for them; whether they have talked with their provider about PSA testing and engaged in an informed decision-making process around prostate cancer screening. We hypothesized that: H1: African American men who have more decision self-efficacy and less decisional conflict about prostate cancer screening will be more likely to have been screened; and H2: African American men who are more knowledgeable about prostate cancer will be more likely to have been screened.

## 2 Methods

Data are from the African American Men’s Health Survey, a cross-sectional study among male, African American patients seen at Augusta University Health. Non-institutionalized men were eligible to take part in the study if they were at least 40 years of age and resided in Augusta-Richmond County or Columbia County, Georgia, or in Aiken County, South Carolina.

The patients were identified using electronic medical records. Data were collected using postal survey questionnaires. The mailings were sent to 1,000 randomly sampled potential research participants. A sequential mailing protocol was followed using a modified Dillman method [8]. An advance letter was mailed to the men by the study principal investigator (SSC). The letter provided information about the study (purpose, potential benefits, and risks). Three weeks later, a survey consent letter (Appendix) was mailed to those who had not opted out along with a copy of the survey questionnaire (Appendix) and a pre-addressed, stamped return envelope. Those who had not opted out or returned a completed questionnaire were sent a reminder postcard three weeks later.

Outcome measures: Information about prostate cancer screening was collected via postal survey. The subjects were asked: Have you ever had a PSA test? (yes / no) and whether they had a PSA test in the past year. They were also asked Do you have a regular health care provider (*e.g.*, doctor, nurse practitioner, physician assistant)? (yes / no) Has a health care provider such as a doctor or nurse ever talked to you about a PSA test? (yes / no) Questions also assessed men’s self-reported levels of prostate cancer knowledge, decision self-efficacy, decisional conflict, control preferences, stage of decision making, and perceived risk related to prostate cancer screening using established reliable scales and measures [9–11]. Decisional conflict was determined by an existing 10-item Likert-type scale [9]. Stage of decision making was determined by one question with six response options to measure different stages of making a decision about the PSA test in the next 12 months, with higher values indicating greater certainty in making a decision about screening (whether to receive it or not).

Descriptive analyses and logistic regression methods were used to examine predictors of prostate cancer screening. In bivariate analyses, levels of statistical significance were determined using the one-sided Wilcoxon rank-sum test. We considered α = 0.05 as the level of statistical significance. Levels of statistical significance were determined using Wald chi-square tests and Log-likelihood ratio tests. We present adjusted odds ratios (OR) and 95% confidence Intervals (95% CI) from logistic regression analyses. The goodness-of-fit of the model model was examined using the Log-likelihood ratio test. The study was approved by the Augusta University Institutional Review Board.

## 3 Results

A total of 65 men completed the study questions (response rate = 6.5%). The mean age of the men was 64.4 years (Table 1). Of the 65 surveyed participants, the majority of participants had two persons living within a household (n = 35, 59.3%), were retired (n = 28, 46.7%), were married or with partner (n = 41, 65.1%), had a HS educational level (n = 16, 25.4%), and reported good general health (n = 33, 53.4%).

As shown in Table 2, most of the participants (90.8%) reported a regular healthcare provider and that their provider had discussed the PSA test with them (81.3%). About 84.1% of the men had had a PSA test, but only 38.0% had one in the past year. About one-fifth of the men (21.9%) had a positive family history of prostate cancer. Most of the men reported that they make the final decision about whether to have a PSA test on their own (36.5%) or after seriously considering their doctor’s opinion (28.6%). About 31.8% of the men reported that they share responsibility about whether to have a PSA test with their doctor. About half of the participants (49.2%) reported that they have made a decision about whether to have a PSA test and they are not likely to change their mind. The majority of the men (75%) perceived their risk of prostate cancer to be about the same level of risk as other men who were their age. The men’s knowledge of prostate cancer was fair to good (mean prostate cancer knowledge scale = 10.37, SD 1.87).

Additional analyzes were performed to examine factors associated with prostate cancer knowledge (results not shown). Knowledge of prostate cancer was positively associated with receipt of a PSA test (p < 0.0206). No significant associations were observed between decision self-efficacy or decisional conflict and receipt of a PSA test (p > 0.05).

When logistic regression models were fitted to the data, only one of the covariates show a significant association with the subject’s history of ever taking a PSA test – the odds of the subjects taking a PSA test increase significantly when their health care provider talked about the PSA test (Table 3). The odds ratio with and without talking about the PSA test is 56.4, with a 95% confidence interval of 7.5-773.7. Among participants whose healthcare provider talked about the PSA test, 50 out of 52 (96.1%) had a PSA test. For participants whose healthcare provider never talked about a PSA test, only 3 out of 11 (27.3%) had a PSA test.

## 4 Discussion

In a survey sample among 65 African American men from the south, we observed a high prevalence (84.1%) of men that have been screened for prostate cancer. A number of reasons have been noted in the literature for the prostate cancer disparity among African American men including inadequate knowledge about prostate cancer; presentation at a later stage of prostate cancer, and more aggressive tumors in African Americans (12-14). In the current study, the majority of the participants (81.3%) reported that a health care provider such as a doctor or nurse had talked to them about a PSA test, and few of the men indicated that their health care provider had failed to encourage them to ask questions or express any concerns they had about PSA testing. Woods-Burnham *et al*. [1] found that less than half of African American men engage in conversations about prostate cancer with a healthcare provider. The U.S. Preventive Services Task Force and the American Cancer Society recommend that men engage in informed decision making with their healthcare provider after learning about the benefits and harms of prostate cancer screening [15,16]. Potential barriers to informed decision making about PSA testing are patient-related (*e.g.*, decreased self-efficacy, fear, medical distrust) and physician-related (*e.g.*, limited availability, lack of knowledge, subpar interpersonal skills) [17]. Potential barriers to prostate cancer screening include lack of health insurance and poorer access to health care [18].

Many of the respondents in the current study had modest knowledge of prostate cancer and prostate cancer screening. Knowledge of prostate cancer has been positively associated with prostate cancer screening in some but not all studies of African American men [18,19]. Patient’s lack of knowledge about prostate cancer and medical recommendations about prostate cancer screening may be a barrier to making an informed decision about PSA testing [19, 20]. A systematic review of 33 papers examined knowledge, awareness, and beliefs about prostate cancer and prostate cancer screening; knowledge of prostate cancer risk, symptoms, diagnostic methods and treatment options were found to contribute to greater willingness to be screened for prostate cancer [21]. Older and low-income African American study participants tend to have less knowledge about prostate cancer, risk factors, and prostate cancer screening than their white counterparts [22,23]. In addition to younger age and higher income, greater educational attainment may be positively associated with knowledge about the prostate gland and prostate cancer screening tests [20,24].

The three major risk factors for prostate cancer are age, race, and family history [19]. Compared to other men their age, few (1.7%) of the men in the current study perceived their risk of prostate cancer to be high or a little high. In an analysis of data from the 2003 Health Information National Trends Study, only 18% of African American men perceived themselves to be more likely to get prostate cancer than the average man of the same age, despite statistics to the contrary [25].

With respect to limitations, misclassification bias is a possibility due to the use of self-reported information. In addition, participation bias may also influence the interpretations of these results as men had to mail in their responses. Furthermore, the results of this study may not be generalizable to other populations of African American men. However, even with the limited sample size, we observed that the sample was diverse across socioeconomic factors including income and education.

In conclusion, the low overall prostate cancer knowledge among these participants, including their risk for prostate cancer, indicates a need for prostate cancer educational interventions in this patient population.

## Supplementary Material

1

## Figures and Tables

**Table 1:** Characteristics of study participants, African American Men’s Health Survey (n = 65)

Characteristic	All n (%)
**Mean age** (SD)* (N = 58)	64.44 (9.32)
**Annual Income** (N = 60)	
< $20,000	9 (15.0)
$20,000 – $34,999	4 (6.7)
$35,000 - $49,999	8 (13.3)
$50,000 - $64,999	9 (15.0)
$65,000 - $79,999	11 (18.3)
$80,000+	8 (13.3)
Missing	11 (18.3)
**Number of people in household** (N = 59)	
1	13 (22.0)
2	35 (59.3)
3+	11 (18.7)
**Employment status** (N = 60)	
Retired	28 (46.7)
Employed	10 (16.7)
On disability	16 (26.7)
Temporarily unemployed	6 (10.0)
**Marital status** (N = 63)	
Married/Partner	41 (65.1)
Single	12 (19.1)
Widowed	3 (4.8)
Separated/Divorced	7 (11.1)
**Education** (N = 63)	
Less than HS	8 (12.7)
HS or equivalent	16 (25.4)
Some college	15 (23.8)
Associate degree	7 (11.1)
Bachelor degree	9 (14.3)
Graduate degree	8 (12.7)
**Perceived general health** (N = 63)	
Excellent	1 (1.6)
Very good	8 (12.7)
Good	33 (52.4)
Fair	16 (25.4)
Poor	5 (7.9)

* **Notes**: SD: standard deviation

**Table 2:** Health characteristics and study variables among African American men seen at Augusta University Health (n = 65)

Characteristic	n (%)
**Have a regular healthcare provider (N = 65)**	59 (90.8)
**Healthcare provider discussed PSA test (N = 64)**	52 (81.3)
**Ever had a PSA test (N = 63)**	53 (84.1)
**Had a PSA test in the past year (N = 50)**	19 (38.0)
**Family history of prostate cancer (N = 64)**	14 (21.9)
**Control preferences (N = 63)**	
I make the final decision on my own	23 (36.5)
I made a decision after seriously considering my doctor’s opinion	18 (28.6)
My doctor and I share responsibility for the decision	20 (31.8)
I prefer that the doctor make the decision after seriously considering my opinion	1 (1.6)
I prefer that the doctor make the decision	1 (1.6)
**Stage of decision-making scale (N = 61)**	
I haven’t thought about it	10 (16.4)
I haven’t thought about it, but I am interested in learning more	9 (14.8)
I have started to think about it, but I haven’t made a decision	6 (9.8)
I have thought about it and I am close to making a decision	3 (4.9)
I have made a decision, but I am willing to reconsider	3 (4.9)
I have made a decision and I am not likely to change my mind	30 (49.2)
**Perceived risk of prostate cancer scale (N = 60)**	
Much lower risk	7 (11.7)
A little lower risk	7 (11.7)
About the same level of risk	45 (75.0)
A little higher risk	1 (.17)
**Knowledge About Prostate Cancer**	Correct Response
Most men diagnosed as having prostate cancer die of something else (N = 60)	28 (46.7)
Men are more likely to die because of prostate cancer than because of heart disease (N = 59)	45 (76.3)
It is possible to have prostate cancer if a man dos not have any symptoms (N = 60)	53 (88.3)
Prostate cancer is one of the least common cancers among men (N = 59)	42 (71.2)
If you have an abnormal PSA test result, your doctor may recommend that you have a prostate biopsy (N = 60)	56 (93.3)
The PSA test will find all prostate cancers (N = 60)	44 (73.3)
A prostate biopsy can tell you with more certainty whether you have prostate cancer than a PSA test (N = 59)	53 (89.8)
Loss of sexual function is a possible side effect of prostate cancer treatments (N = 60)	51 (85.0)
Problems with urination are possible side effects of prostate cancer treatments (N = 59)	49 (83.1)
The risk of developing prostate cancer increases with age (N = 61)	55 (90.2)
The risk of developing prostate cancer is higher in African American men as compared with men from other racial/ethnic groups (N = 56)	50 (89.3)
The risk of developing prostate cancer increases if you have a father or brother who has had prostate cancer (N = 61)	46 (75.4)
Diet rich in fruits is likely to reduce risk for developing prostate cancer (N = 60)	38 (63.3)
	Mean (SD)
**Decision self-efficacy (N = 56)**	38.98 (6.79)
**Decisional conflict (N = 55)**	33.93 (4.98)

**Notes**: N: the total sample. f: the frequency or correct response for Knowledge About Prostate Cancer questions (%: the relative frequency).

**Table 3:** ORs and associated 95% CIs from univariable logistic regression models and associated p-values to examine association of different covariates with the subject taking a PSA test

Characteristic	OR (95% CI)	p-value	Overall p-value
**Age (continuous)**	1.03 (0.95 – 1.13)	0.491	0.491
**Annual Income**			
< $20,000 (Referent)	1.00		0.325
$20,000 – $34,999	1.00(0.06 – 26.86)	1.000	
$35,000 - $49,999	2.33 (0.18 – 58.01)	0.529	
$50,000 - $64,999	Undefined	-	
$65,000 - $79,999	0.58 (0.06 – 4.19)	0.601	
$80,000+	Undefined	-	
**Education**			
Less than HS (Referent)	1.00		0.609
HS or equivalent	2.60 (0.26 – 27.19)	0.398	
Some college	2.60 (0.26 – 27.19)	0.398	
Associate degree	1.00 (0.09 – 11.32)	1.000	
Bachelor degree	Undefined	0.994	
Graduate degree	2.8 (0.21 – 70.84)	0.448	
**History of prostate cancer in immediate family**	3.00 (0.49 – 58.02)	0.307	0.307
**Health care provider talked about a PSA test**	66.77 (11.47 – 618.94)	< 0.001	< 0.001
**Perceived risk of prostate cancer scale**			
Much lower risk (Referent)	1.00		0.521
A little lower risk	Undefined	0.994	
About the same level of risk	Undefined	0.994	
A little higher risk	Undefined	1.000	
**Prostate cancer knowledge scale**	1.32 (0.88 – 1.99)	0.169	0.169
**Decisional conflict**	1.02 (0.88 – 1.21)	0.775	0.775
**Decision self-efficacy**	0.96 (0.82 – 1.02)	0.493	0.493

**Notes**: Undefined denotes estimates either with extreme/infinity values or too small.
