# Supplementary material for: Predictors of prostate cancer screening among African American men treated at an Academic Medical Center in the Southern United States
Source: Curr Cancer Rep. Author manuscript; Available in PMC 2021 Jun 15. (PMC8064558; doi:10.25082/CCR.2021.01.003)
Supplement: 1 [file NIHMS1690168-supplement-1.pdf]

Appendix

A Consent Letter

Dear xxx,

Researchers at Augusta University are conducting a health survey of African American men by mail. The survey asks questions about health status, medical history, prostate cancer screening, and smoking. It should take you about 45 minutes to complete. The results of this study will be used to obtain scientific information about the health of African American men who reside in Augusta and surrounding areas of Georgia and South Carolina. Through your participation we hope to understand the health of African American men so that prevention and treatment efforts can be improved. Results from the study will be published in scientific journals and presented at scientific conferences.

There are no known risks to you if you decide to participate in this survey other than potential, minor psychological distress. Some of the questions deal with sensitive subjects, including your physical and mental health, so we can get a more complete understanding of your health. Some people get distressed when answering these types of questions. There is no direct benefit to you for participating in this study. You will not be compensated for your participation in this research. I will not share any information that identifies you with anyone outside my research group at Augusta University.

This survey is voluntary and there is no penalty if you do not participate. It's up to you whether to answer the survey. You can skip any questions that you don't want to answer. If you do not participate, your decision will not adversely affect your relationship with Augusta University, if any. If you decide to take part in this study, you will be contacted in 4 to 5 years for a follow-up survey so that we can learn about changes in health over time. You may also receive information about additional health studies you may be eligible to participate in.

The data you provide will be stored at Augusta University. Your name and contact information will be stored separately in a locked cabinet for approximately 4 to 5 years. The data will be made available to other researchers for other studies following the completion of this research study and will not contain information that could identify you.

I hope you will take the time to complete this survey. A pre-addressed, stamped return envelope is enclosed for your convenience. If you have any questions or concerns about completing survey, about being in this study, or to receive a summary of my findings you may contact me at (706) 721-2270. If you have any questions or concerns about the "rights of research subjects", you may contact the Augusta University IRB Office at (706) 721-1483.

Sincerely,

Steven S. Coughlin, PhD
Professor of Epidemiology
Department of Population Health Sciences
Medical College of Georgia
Augusta University
1120 15th Street, AE-1042
Augusta, GA 30912

B African American men's health survey

QUESTIONNAIRE

MARKING INSTRUCTIONS

- While you can use a pen, please use a PENCIL in case you want to change an answer.
- Please do NOT use felt tip pens.
- Make solid, heavy "X" marks in the box.
- Please erase cleanly any mark you wish to change.
- Please do not make any stray marks on this form.

(This page will be kept separately from the rest of the pages to protect your privacy)

PLEASE PRINT

Name: ______________ (First) ______________ (Middle) ______________ (Last)
Address: ______________ (City) ______________ (State) ______________ (Zip Code)
Best telephone numbers to reach you at: (_______) ______________-______________
☐ Cellular ☐ Home ☐ Work
(_______) ______________-______________
☐ Cellular ☐ Home ☐ Work
Email address: ____________________________

Before you begin the survey, please respond to the following statements.

1. I am 40 years of age or older
a. Yes
b. No
2. I live in Augusta-Richmond County or surrounding areas of Georgia or South Carolina.

a. Yes
b. No

If you answered No to either statement above, you do not need to complete the rest of the survey. Please return the survey in the postage paid envelope. We thank you for your time.

If you answered Yes to each statement above, you should complete the full survey.

PLEASE START HERE

3. In general, would you say your overall health is:
☐ 1- Excellent
☐ 2- Very good
☐ 3- Good
☐ 4- Fair
☐ 5- Poor

4. How much **bodily** pain have you had **during the past 4 weeks**:
☐ 1- None
☐ 2- Very mild
☐ 3- Mild
☐ 4- Moderate
☐ 5- Severe
☐ 6- Very Severe

5. For how long (if at all) has your **health limited you** in **each** of the following activities?

	Limited for more than 3 months	Limited for 3 months or less	Not limited at all
a. The kinds or amounts or vigorous activities you can do, like lifting heavy objects, running or participating in strenuous sports	0 – 1	0 – 2	0 – 3
b. The kind or amounts of moderate activities you can do, like moving a table, carrying groceries, or bowling	0 – 1	0 – 2	0 – 3
c. Walking uphill or climbing a few flights of stairs	0 – 1	0 – 2	0 – 3
d. Bending, lifting, or stooping	0 – 1	0 – 2	0 – 3
e. Walking one block	0 – 1	0 – 2	0 – 3
f. Eating, dressing, bathing, or using the toilet	0 – 1	0 – 2	0 – 3

6. Does your health **keep** you from working at a job, doing work around the house, or going to school?
☐ 1- YES, for more than 3 months
☐ 2- YES, for 3 months or less
☐ 3- NO

7. Have you been unable to do **certain kinds or amounts** of work, housework, or schoolwork because of your health?
☐ 1- YES, for more than 3 months
☐ 2- YES, for 3 months or less
☐ 3- NO

For **each** of the following questions, please mark the circle for the **one** answer that comes **closest** to the way you have been feeling **during the past month**.

	All of the time	Most of the time	A good bit of the time	Some of the time	A little of the time	None of the time
8. How much of the time, during the past month, has your **health limited your social activities** (like visiting with friends or close relatives)?	☐ 1	☐ 2	☐ 3	☐ 4	☐ 5	☐ 6
9. How much of the time, during the past month, have you been a very nervous person?	☐ 1	☐ 2	☐ 3	☐ 4	☐ 5	☐ 6
10. During the past month, how much of the time have you felt calm and peaceful?	☐ 1	☐ 2	☐ 3	☐ 4	☐ 5	☐ 6
11. How much of the time, during the past month, have you felt downhearted and blue?	☐ 1	☐ 2	☐ 3	☐ 4	☐ 5	☐ 6
12. During the past month, how much of the time have you been a happy person?	☐ 1	☐ 2	☐ 3	☐ 4	☐ 5	☐ 6
13. How often, during the past month, have you felt so down in the dumps that nothing could cheer you up?	☐ 1	☐ 2	☐ 3	☐ 4	☐ 5	☐ 6

14. Please mark the square that best describes whether **each** of the following statements is true or false for you.

	Definitely true	Mostly true	Not sure	Mostly false
a. I am somewhat ill	☐ 1	☐ 2	☐ 3	☐ 4
b. I am as healthy as anybody I know	☐ 1	☐ 2	☐ 3	☐ 4
c. My health is excellent	☐ 1	☐ 2	☐ 3	☐ 4
d. I have been feeling bad lately	☐ 1	☐ 2	☐ 3	☐ 4

15. Do you drink alcohol on a regular basis (irrespective of amount of alcohol consumed)?
a. Yes
b. No
c. Prefer not to answer

HEALTH INFORMATION

Please mark an X beside each of the health conditions that you discussed with a healthcare provider and indicate the date (month/year) it was first noted.

☐ High blood pressure
☐ High cholesterol
☐ Stomach problem
☐ Lung/breathing problem
☐ Rheumatoid arthritis
☐ Congestive heart failure
☐ Heart attack
☐ Stroke
☐ Chronic bronchitis
☐ Emphysema
☐ Asthma
☐ Bladder/kidney problem
☐ Depression
☐ Anxiety
☐ Diabetes/sugar problem
☐ Osteoporosis
☐ Cancer (Specify type) ______________________________
☐ Other ______________________________

Are you currently taking medications for any of the conditions below? Please CHECK ALL that apply

☐ Diabetes
☐ High blood pressure
☐ High cholesterol
☐ Other (Please specify)______________________________

Do you have any other condition or illness that was not checked above?
If so, what is it? ______________________________

PROSTATE CANCER SCREENING

16. Have you ever heard of a PSA or prostate-specific antigen test?
a) yes
b) no
17. Have you ever had a PSA test?
a) yes
b) no (**skip to 19**)
18. When did you have your most recent PSA test?
a) a year ago or less
b) more than 1 but not more than 2 years ago
c) more than 2 but not more than 5 years ago
d) over 5 years ago

The next few questions are about discussions that health care providers might have had with you about the PSA test.

19. Do you have a regular health care provider (e.g., doctor, nurse practitioner, PA)?
a) yes
b) no
20. Has a health care provider such as a doctor or nurse ever talked to you about a PSA test?
a) yes
b) no (**skip to 23**)
21. Thinking about the last time a health care provider talked to you about a PSA test, which of the following statements best describes your health care provider's recommendation about PSA tests?
a) that you should have a PSA test
b) that you should not have a PSA test
c) your health care provider did not make a recommendation
22. Thinking about the last time a health care provider talked to you about a PSA test, did your health care provider encourage you to ask questions or express any concerns you had about PSA testing? Would you say . . .
a) yes, definitely
b) yes, somewhat, or
c) no, not at all
d) DID NOT HAVE ANY QUESTIONS OR CONCERNS ABOUT PSA

<u>My Confidence in making an informed choice</u> **Decision Self-Efficacy Scale**

Below are listed some things involved in making an informed choice. Please show how confident you feel in doing these things by circling the number from 0 (not at all confident about PSA testing or not getting it) to 4 (very confident) for each item below.

I feel confident that I can:

23. Get the facts about the prostate cancer screening decisions choices available to me	Not at all confident	Somewhat not confident	Neutral	Somewhat confident	Very confident
24. Get the facts about the benefits of each choic	Not at all confident	Somewhat not confident	Neutral	Somewhat confident	Very confident
25. Get the facts about the risks and side effects of each choice, whether or not to get prostate cancer screening	Not at all confident	Somewhat not confident	Neutral	Somewhat confident	Very confident
26. Understand the information enough to be able to make a choice	Not at all confident	Somewhat not confident	Neutral	Somewhat confident	Very confident
27. Ask questions without feeling dumb	Not at all confident	Somewhat not confident	Neutral	Somewhat confident	Very confident
28. Express my concerns about each choice	Not at all confident	Somewhat not confident	Neutral	Somewhat confident	Very confident
29. Ask for advice	Not at all confident	Somewhat not confident	Neutral	Somewhat confident	Very confident
30. Figure out the choice that best suits me	Not at all confident	Somewhat not confident	Neutral	Somewhat confident	Very confident
31. Handle unwanted pressure from others in making my choice	Not at all confident	Somewhat not confident	Neutral	Somewhat confident	Very confident
32. Let my healthcare provider know what's best for me	Not at all confident	Somewhat not confident	Neutral	Somewhat confident	Very confident
33. Delay my decision if I feel I need more time	Not at all confident	Somewhat not confident	Neutral	Somewhat confident	Very confident

Please answer the following questions about how confident you are about making an informed decision about prostate cancer screening: ***Decisional Conflict Scale***

34. I'm aware of the choices I have for prostate cancer screening.	Strongly disagree	Disagree	Agree	Strongly agree
35. I feel I know the benefits of prostate cancer screening.	Strongly disagree	Disagree	Agree	Strongly agree
36. I feel I know the risks and side effects of prostate cancer screening.	Strongly disagree	Disagree	Agree	Strongly agree
37. I know how important the benefits are for prostate cancer screening.	Strongly disagree	Disagree	Agree	Strongly agree
38. I am clear about which risks and side effects matter most to me for prostate cancer screening.	Strongly disagree	Disagree	Agree	Strongly agree
39. I have enough support from others to make a choice.	Strongly disagree	Disagree	Agree	Strongly agree
40. I choose without pressure from others.	Strongly disagree	Disagree	Agree	Strongly agree
41. I have enough advice to make an informed choice.	Strongly disagree	Disagree	Agree	Strongly agree
42. I am satisfied with my decision.	Strongly disagree	Disagree	Agree	Strongly agree
43. I expect to stick with my decision.	Strongly disagree	Disagree	Agree	Strongly agree

We are interested in how satisfied you are with your decision about prostate cancer screening. Please indicate how much you agree or disagree with the following statements ***Satisfaction with Decision Scale (post-test only)***

44. I am satisfied that I was adequately informed about the issues important to my decision about screening for prostate cancer.	Strongly disagree	Disagree	Agree	Strongly agree
45. The decision I made about prostate cancer screening was the best decision possible for me personally.	Strongly disagree	Disagree	Agree	Strongly agree
46. I am satisfied that my decision about prostate cancer screening was consistent with my personal values.	Strongly disagree	Disagree	Agree	Strongly agree
47. I expect to continue to carry out the decision I made about prostate cancer screening.	Strongly disagree	Disagree	Agree	Strongly agree
48. I am satisfied that this was my decision to make.	Strongly disagree	Disagree	Agree	Strongly agree
49. I am satisfied with my decision about prostate cancer screening.	Strongly disagree	Disagree	Agree	Strongly agree

50. Who should make medical decisions? **Control Preferences Scale**
a) I make the final decision on my own
b) I made a decision after seriously considering my doctor's opinion
c) My doctor and I share responsibility for the decision
d) I prefer that the doctor make the decision after seriously considering my opinion
e) I prefer that the doctor make the decision
51. When you think about getting a PSA test in the next 12 months, which sentence best describes you? **Stage of Decision Making Scale**
a) I haven't thought about it
b) I haven't thought about it, but I am interested in learning more
c) I have started to think about it, but I haven't made a decision
d) I have thought about it and I am close to making a decision
e) I have made a decision, but I am willing to reconsider
f) I have made a decision and I am not likely to change my mind

52. What do you think is your risk of developing prostate cancer compared to other men your age? **Perceived Risk of Prostate Cancer Scale** a) Much lower risk
b) A little lower risk
c) About the same level of risk
d) A little higher risk
e) Much higher risk
My Knowledge about Prostate Cancer **Prostate Cancer Knowledge Scale**
53. Most men diagnosed as having prostate cancer die of something else _______________
54. Men are more likely to die because of prostate cancer than because of heart disease _______________
55. It is possible to have prostate cancer if a man does not have any symptoms _______________
56. Prostate cancer is one of the least common cancers among men _______________
57. If you have an abnormal PSA test result, your doctor may recommend that you have a prostate biopsy _______________
58. The PSA test will find all prostate cancers _______________
59. A prostate biopsy can tell you with more certainty whether you have prostate cancer than a PSA test _______________
60. Loss of sexual function is a possible side effect of prostate cancer treatments _______________
61. Problems with urination are possible side effects of prostate cancer treatments _______________
62. The risk of developing prostate cancer increases with age _______________
63. The risk of developing prostate cancer is higher in African American men as compared with men from other racial/ethnic groups _______________
64. The risk of developing prostate cancer increases if you have a father or brother who has had prostate cancer _______________
65. Diet rich in fruits is likely to reduce risk for developing prostate cancer _______________
66. Do you have a history of prostate cancer in your immediate family (such as a father, brother)?
a) yes
b) no
67. What is your height? (please enter your height in feet and inches)
________ ________ ________
Q68. How much do you weigh? (Please enter your weight in pounds)
________ ________ ________
Q69. How do you describe your weight? (Circle correct answer)
a. Very underweight
b. Slightly underweight
c. About the right weight
d. Slightly overweight
e. Very overweight
f. Prefer not to answer
Q70. Which are the following are you trying to do about your weight? (Circle correct answer)
a. Lose weight
b. Stay the same
c. Gain weight
d. Not trying to do anything about my weight
e. Prefer not to answer

TOBACCO USE

Q71. Have you ever smoked a cigarette?
a. Yes
b. No
Q72. If answered yes on Q71, Have you smoked 100 cigarettes (5 packs) in your lifetime?
c. Yes
d. No
Q73. If answered yes on Q71. Do you now smoke cigarettes?
a. Every day
c. Some days
d. Not at all
Q74. If answered yes on Q71. How old were you the first time you smoked part or all of a cigarette?
OR
Q74. If answered yes on Q71. How old were you when you first started smoking fairly regularly?
Q75. How many cigarettes smoked per day when you smoked fairly regularly ____________________
Q.76. [On the days that you smoke] How soon after you wake up do you typically smoke your first cigarette of the day? Please enter the number of minutes or hours ______________________________
Q.77. In the past 12 months, how many times have you stopped smoking for one day or longer because you were trying to quit?
a. 0 times
b. 1 time
c. 2-3 times
d. 4 or more times
Q78. When do you plan to quit smoking for good?
a. In the next 7 days
b. In the next 30 days
c. In the next 6 months
d. In the next year

e. More than one year from now
f. I never plan to quit smoking

E-cigarette use

Q79. Have you ever used Vaporizers, E-Cigarettes, and other Electronic Nicotine Delivery Systems (ENDS) some brand examples include JUUL, NJOY, Blu, Vuse, MarkTen, Logic, Vapin Plus, eGo, Halo, GreenSmoke, Fin, and KangerTech.
Yes
No
Q80. Have you ever used Vaporizers, E-Cigarettes, and other ENDS fairly regularly?
Yes
No
Q81. Do you now use Vaporizers, E-Cigarettes, and other ENDS?
Everyday
Somedays
Not at all
Q82. Have you ever smoked little filtered cigars or cigarillos, some brand names include Black and Mild, White Owl, and Swisher Sweets?
Yes
No
Q83. Have you ever smoked little filtered cigars or cigarillos, some brand names include Black and Mild, White Owl, and Swisher Sweets fairly regularly?
Yes
No
Q84. Do you now smoke little filtered cigars or cigarillos, some brand names include Black and Mild, White Owl, and Swisher Sweets?
Everyday
Somedays
Not at all
Q85. Have you ever used Smokeless Tobacco Products, Including Dip, Snuff, Snus, and Chewing Tobacco?
Yes
No
Q86. Have you ever used Smokeless Tobacco Products, Including Dip, Snuff, Snus, and Chewing Tobacco fairly regularly?
Yes
No
Q87. Do you now use Smokeless Tobacco Products, Including Dip, Snuff, Snus, and Chewing Tobacco?
Everyday
Someday
Not at all

E-cigarettes/ENDS are considered tobacco products by the FDA because most of them contain nicotine, which comes from tobacco. There are increasing concerns about the health risks associated with use of e-cigarettes/ENDS. You can find out more about these issues by clicking on the Advisory on E-Cigarette Use among Youth issued by the U.S. Surgeon General, and the Severe Pulmonary Disease Associated with Using E-Cigarette products issued by the Centers for Disease Control & Prevention (CDC).

DEMOGRAPHICS

88. Age: ______________ years
89. Race/Ethnicity
a) African American
b) Afro-Caribbean
c) Afro-Haitian
d) Afro-Hispanic
e) African
f) Other
90. Marital status:
a. Single
b. Married
c. Partner
d. Separated
e. Divorced
f. Widowed
g. I choose not to answer
91. Education
f. Less than High School degree
g. High School Degree or equivalent (e.g., GED)
h. Some College but no degree
i. Associate Degree
j. Bachelor Degree
k. Graduate Degree
l. I choose not to answer
92. Annual household income

m. < $20,000
n. $20,000 - $34,999
o. $35,000 - $49,999
p. $50,000 - $64,999
q. $65,000 - $79,999
r. $80,000 to $99,00
s. $100,000 or more
t. I choose not to answer
u. I don't know
93. Number of people in household: ______________________________
94. Employment status:
v. Temporarily unemployed
w. Employed (> 20 hours / week)
x. Homemaker
y. On disability
z. Retired

That's all the questions we have for you. Thank you for your time.

Current Cancer Reports © 2021 by SyncSci Publishing & All rights reserved
